# Motivators, Barriers, and Facilitators to Traveling to the Safest Hospitals in the United States for Complex Cancer Surgery

**DOI:** 10.1001/jamanetworkopen.2018.4595

**Published:** 2018-11-16

**Authors:** Benjamin J. Resio, Alexander S. Chiu, Jessica R. Hoag, Lawrence B. Brown, Marney White, Audry Omar, Andres Monsalve, Andrew P. Dhanasopon, Justin D. Blasberg, Daniel J. Boffa

**Affiliations:** 1 Section of Thoracic Surgery, Department of Surgery, Yale School of Medicine, New Haven, Connecticut; 2 Yale School of Public Health, New Haven, Connecticut; 3 Yale Center for Analytical Sciences, New Haven, Connecticut

## Abstract

**Importance:**

Directing patients to safer hospitals for complex cancer surgery (regionalization) may prevent thousands of mortalities in the United States.

**Objective:**

To understand the potential for individuals to move to safer hospitals: what would inspire them to travel (motivators), what challenges would they face (barriers), and what would enable them to travel (facilitators).

**Design, Setting, and Participants:**

This nationally representative online survey study asked respondents to consider complex cancer surgery at their local hospital or a hospital specializing in cancer an hour farther away. Completed surveys were weighted across sociodemographics to be nationally representative and outcomes were reported as weighted percentages. In January 2018, a panel of 1817 US adults recruited by address- and telephone-based sampling to be nationally representative were invited to take the survey. Data analysis was conducted from January 24, 2018, to September 19, 2018.

**Main Outcomes and Measures:**

Proportion of respondents motivated to travel by specific quality and safety indicators (motivators), magnitude in difference that would be necessary, proportion facing specific barriers, and proportion enabled to move by facilitators. Resistant individuals were identified as people who would not travel except for the largest (top quartile) outcomes differences.

**Results:**

There were 1016 completed surveys (response rate of 55.9%). The weighted median age was 48 years, 52% were female, median annual income was between $60 000 and $75 000, and 85% lived in a metropolitan area. Nonresponders were more likely than responders to be female, younger, nonwhite, less educated, and lower income (female: 54.4% vs 48.3%; *P* = .01; younger [aged <45 years]: 56.3% vs 37.1%; *P* < .001; nonwhite: 41.6% vs 30.0%; *P* < .001; less than college education: 43.8% vs 32.4%; P < .001; income <$30 000: 22.1% vs 17.1%; *P* = .01). Superior safety or oncologic outcomes, presented separately, motivated an average of 92% of respondents (95% CI, 90%-94%) to travel. One-third were easily motivated, requiring less than 1% advantage in safety or quality, while 12% were particularly resistant across outcomes. Respondents with lower income (income <$25 000: odds ratio, 2.01; 95% CI, 1.19-3.39) and nonwhite race (odds ratio, 1.60; 95% CI, 1.05-2.42) were more resistant to travel. At least 1 barrier was identified by 74% of respondents (95% CI, 72%-77%), most commonly financial (costs/insurance). However, 94% of respondents (95% CI, 92%-96%) with barriers would travel if provided facilitators, many of which were relatively low cost (transportation, parking, and hotel).

**Conclusions and Relevance:**

It appears that most of the US public could be motivated to travel to safer hospitals for complex cancer surgery, yet most would require some support to move. Further efforts to ensure that benefits from regionalization are equitable across sociodemographic strata are indicated.

## Key Points

**Question:**

What information may motivate the US public to travel to safer hospitals for complex cancer surgery, what barriers to traveling do they face, and what solutions may facilitate appropriately changing hospitals?

**Findings:**

In this nationally representative survey study, 92% of respondents would be motivated to travel to a specialty cancer hospital for superior safety or oncologic outcomes, but 74% also reported barriers to traveling, although most of the barriers could be overcome with proposed solutions. Specific socioeconomic subsets were less likely to travel.

**Meaning:**

It appears that most of the US public could be motivated to travel to safer hospitals for complex cancer surgery, yet most would require some support to move. Further efforts to ensure that benefits from regionalization are equitable across sociodemographic strata are indicated.

## Teaser

This nationally representative survey study examines the motivators, barriers, and facilitators for traveling to the safest hospitals in the United States for patients needing complex cancer surgery.

## Introduction

The risk of lethal complications after complex cancer surgery varies tremendously across hospitals in the United States, with some of the safest hospitals experiencing half the surgical mortality rates of the less safe hospitals.^1,2,3,4,5^ In fact, it is estimated that over 1000 deaths could be prevented each year by directing patients to the safest hospitals for complex cancer surgery.^6^ As a result, efforts are under way to minimize the performance of complex cancer surgery at particularly unsafe hospitals (regionalization).^1,4,7,8,9^

However, within decentralized health care systems such as the United States, the effect of any strategy to regionalize health care will rely heavily on the willingness and ability of individuals to travel to specialty hospitals. Because the safest hospitals for most people are not their closest hospitals,^7^ efforts to direct the public to alternative hospitals must take into consideration their potential to travel. It is currently unknown what hospital-level information would inspire people in the United States to travel to a safer hospital (known as *motivators*). In addition, the extent to which motivated persons are prevented from traveling to safer hospitals and the specific challenges they face (known as *barriers*) are unclear. Finally, the types of support that could allow people in the United States to overcome barriers and enable them to travel to safer hospitals for complex cancer surgery (known as *facilitators*) have yet to be identified. In an effort to understand the public perspective of motivators, barriers, and facilitators to traveling to a specialty hospital for complex cancer surgery, we surveyed a nationally representative sample of over 1000 people living in the United States.

## Methods

### Survey Development

A focus group was conducted to inform the development and comprehension of survey questions, leading to a list of candidate motivators, barriers, and facilitators. The final questionnaire was generated and tested in pilot sampling (eAppendix 1 in the Supplement). For each set of questions, respondents were asked to envision a scenario where they had been diagnosed with cancer for which a complex surgery was their best chance of a cure. Two settings were offered for surgery: (1) a smaller hospital close to home, or (2) a hospital that specializes in cancer surgery that is 1 hour farther away than their local hospital. In an effort to understand the types of information that could motivate the US public to travel to a hospital that specializes in cancer surgery, 6 quality and safety indicators favoring the specialty hospital were differentially presented (complication rate, infection rate, mortality rate, cure rate, complete resection rate, and surgeon volume) and respondents indicated their preference for surgery. To understand the barriers to travel, respondents were presented with 13 specific logistical challenges and resource limitations and asked to identify which, if any, would prevent them from traveling 1 hour to the specialty hospital (even if they thought the specialty hospital was superior). Finally, in an effort to understand important facilitators for travel, respondents were presented with 8 specific resources and asked to indicate which, if any, if provided for free, would enable them to travel to the specialty hospital. This study had institutional review board approval through the Yale Human Investigations Committee with consent waived because the use or disclosure of protected health information involved no more than a minimal risk to the privacy of individuals. Response and qualification rates were provided from GfK in accordance with the American Association for Public Opinion Research (AAPOR) reporting guideline.

### Survey Administration

The survey was administered online to a random sample of a nationally representative cohort as determined by a probability-based method.^10^ More specifically, the GfK method attempts to recruit a national profile by random-digit telephone dialing and address-based sampling as previously described.^11,12,13,14^ After participants join the panel they complete a demographic survey, which allows for curation of the sample (surveying and weighing national cohorts).^10^ Panel members are offered incentives such as cash rewards and other prizes to complete surveys. Members receive an email and additional telephone reminders to complete surveys. A nationally representative cohort of panel members selected randomly by GfK was repeatedly contacted via direct email and telephone solicitation until the target number of completed surveys was achieved (in this case >1000).^10^

Completed surveys were weighted to match the US census with respect to age, sex, race and ethnicity, education, census region, household income, home ownership status, and metropolitan area status (unweighted and weighted respondent demographics available in eTable 1 in the Supplement). The survey was distributed to 1817 American adults (aged ≥18 years) in January 2018. Nine surveys were excluded because of incomplete data, resulting in 1016 completed surveys (response rate, 55.9%). In an effort to understand differential response rates across sociodemographic strata, nonresponders were compared with responders. In general, nonresponders were more likely to be female (54.4% vs 48.3%; *P* = .01), younger (aged <45 years: 56.3% vs 37.1%; *P* < .001), nonwhite (41.6% vs 30.0%; *P* < .001), less educated (high school or less: 43.8% vs 32.4%; *P* < .001), and lower income (income less than $30 000: 22.1% vs 17.1%; *P* = .01) (eTable 2 in the Supplement). These trends of nonresponse are consistent with previous US surveys.^15^ Postsurvey weighting was used to account for differential response rates across sociodemographic strata to generate a nationally representative data set.

### Statistical Analysis

Descriptive results were analyzed and presented as weighted percentages of total respondents. Multivariable logistic regression was used to identify groups least likely to travel adjusting for education, residence in a metropolitan statistical area, age, ethnicity, income, sex, previous surgery or cancer diagnosis, and travel time from one’s local hospital. Categorical variables were compared with χ^2^ test and continuous variables with *t* test. Binomial 95% confidence intervals were generated for proportions. Two-sided *P* values less than .05 were considered statistically significant. Analysis was performed in SAS Statistical Software, version 9.4 (SAS Institute Inc).

## Results

### Participants

A total of 1016 surveys were completed (response rate of 55.9%). The weighted median age was 48 years and 52% of participants were female. The median household income bracket was $60 000 to $75 000 annually; 60% had a high school education or higher; and 85% lived in a metropolitan area (Table).

**Table.** Weighted Demographics of Respondents

Variable	Unweighted No. of Respondents (%)	Weighted % of Respondents
No.	1016 (100.0)	
Sex		
Male	525 (51.7)	48.4
Female	491 (48.3)	51.6
Age, y		
18-29	167 (16.4)	21.1
30-44	210 (20.7)	25.0
45-59	277 (27.3)	26.0
≥60	362 (35.6)	27.9
Race/ethnicity		
Non-Hispanic white	716 (70.5)	64.0
Non-Hispanic black	108 (10.6)	11.8
Non-Hispanic other	47 (4.6)	4.8
Hispanic	112 (11.0)	15.9
Non-Hispanic >2 races	33 (3.2)	3.5
Annual household income, $		
<30 000	174 (17.1)	19.2
30 000-59 999	228 (22.4)	22.9
60 000-99 999	321 (31.6)	23.4
100 000-199 999	299 (29.4)	28.2
≥200 000	69 (6.8)	3.4
Metropolitan area resident		
Nonmetropolitan	155 (15.3)	14.6
Metropolitan	861 (84.7)	85.4
US region		
Northeast	165 (16.2)	17.8
Midwest	241 (23.7)	20.8
South	367 (36.1)	37.7
West	243 (24.0)	23.7
Education level		
Less than high school	74 (7.3)	9.6
High school	255 (25.1)	30.3
Some college	292 (28.7)	28.6
Bachelor’s degree or higher	395 (38.9)	31.5
Had surgery in the past		
Yes	512 (50.4)	46.5
No	504 (49.6)	53.6
Had cancer in the past		
Yes	89 (8.8)	7.5
No	927 (91.2)	92.6
Makes own medical decisions		
Yes	848 (83.5)	82.2
No	168 (16.5)	17.8

### Travel Time

The median travel time required by respondents to reach their local hospital was 15 minutes (interquatile range [IQR], 5-25 minutes). The median time that respondents would consider traveling to attend the best hospital for cancer surgery was 120 minutes (IQR, 63-300 minutes). Travel time to a respondent’s local hospital was not correlated with the duration of travel time they would accept to attend the best hospital for surgery, as the Pearson correlation coefficient approached 0 (0.04).

### Motivators to Travel to a Specialty Hospital

Respondents were asked to consider having complex cancer surgery at a smaller local hospital or a specialty hospital that was 1 hour farther away. Six quality and safety indicators were differentially presented favoring the specialty hospital. The effect of all 6 indicators was similar, with superior outcomes at the specialty hospital motivating approximately 92% of respondents (95% CI, 90%-94%) to seek care at the specialty hospital in each category (eTable 3 in the Supplement).

**Figure 1.**
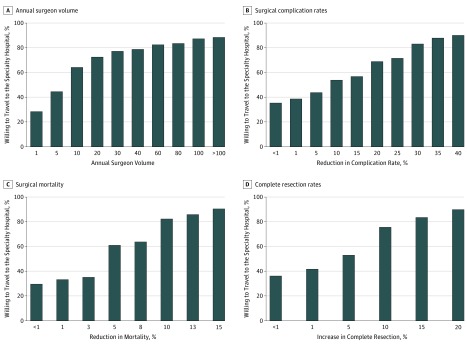
Thresholds to Travel A separate graph is shown for 4 of the 6 quality and safety indicators. Each bar represents the total percentage of who would travel at any given threshold (and therefore includes respondents who indicated a specific threshold or any smaller margin).

In an effort to understand motivators with greater precision, respondents were then asked to define the magnitude of differential in the quality or safety indicator that would motivate them to travel 1 hour to the specialty hospital (Figure 1; eFigures 1 and 2 in the Supplement). Across all 6 indicators, approximately one-third of respondents (30%-38%) appeared to be easily motivated, as they were willing to move with minimal differential (<1%) favoring the specialty hospital. Overall, there was marked variability among individual respondents with respect to thresholds for traveling to the specialty hospital. Forty-five percent of respondents who were in the most easily motivated quartile for at least 1 quality indicator were also in the most resistant quartile (ie, requiring the greatest differential in quality or safety to be motivated to travel) for 1 or more of the other quality indicators.

### Resistant Subset

Resistant respondents (n = 123 [12%]) were defined as individuals who either would not move to the specialty hospital under any circumstances, or required the most dramatic differences (top quartile of respondents) for 5 or more safety or quality motivators before they would travel. For example, respondents who only would move to the specialty hospital if the chance of death was at least 10 percentage points lower than the local hospital (upper quartile of responses) would be considered to be resistant for that motivator. Individuals who were resistant for 5 or more motivators were considered resistant respondents.

Lower income (income ≤$25 000 vs >$25 000 annually: odds ratio [OR], 2.01; 95% CI, 1.19-3.39; *P* = .01), nonwhite race (vs white race: OR, 1.60; 95% CI, 1.05-2.42; *P* = .03), metropolitan residence (vs nonmetropolitan residence: OR, 2.08; 95% CI, 1.01-4.28; *P* = .047), personal history of cancer (OR, 2.07; 95% CI, 1.05-4.07; *P* = .04), and not having received surgery in the past (OR, 1.40; 95% CI, 1.05-1.86; *P* = .004) were associated with being resistant to switching to the specialty hospital (eTable 4 in the Supplement). Respondents aged 35 years or younger were less likely to be in the resistant subset (vs respondents aged 36-50 years: OR, 0.53; 95% CI, 0.28-0.99; *P* = .04).

### Barriers to Traveling to a Specialty Hospital

**Figure 2.**
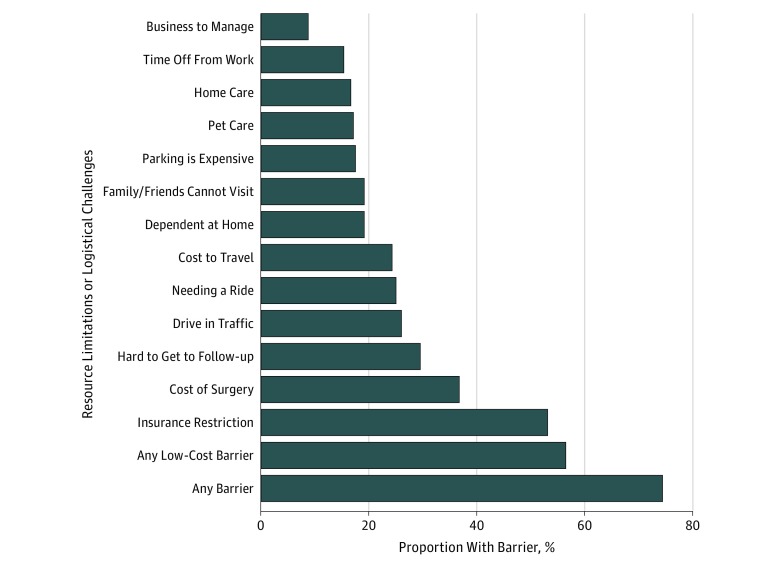
Barriers to Traveling to a Distant Specialty Hospital

Respondents were asked to indicate whether specific logistical challenges and resource limitations (known as barriers) would restrict them from traveling 1 hour to the specialty hospital (Figure 2). At least 1 barrier was noted by 74% of respondents (95% CI, 72%-77%) (31% had 1-2 barriers, 16% had 3-4 barriers, and 27% had ≥5 barriers). Individuals were variably affected by each barrier, as the frequency of restricted persons ranged from 9% to 53% across specific barriers. Financial barriers appeared the most often, including the belief that insurance would restrict them from undergoing surgery at a specialty hospital (53%; 95% CI, 50%-56%) and that surgery would be more expensive (37%; 95% CI, 34%-40%).

### Restricted Subset

Restricted respondents (n = 356) were defined as those with greater than the median number of reported barriers (≥3 barriers). Multivariable logistic regression was performed to identify the attributes of individuals who were particularly restricted from travel. Female (OR, 1.66; 95% CI, 1.25-2.20; *P* < .001) and lower income (income ≤$25 000 vs >$25 000 annually: OR, 2.90; 95% CI, 1.94-4.33; *P* < .001) were significantly associated with having more barriers to travel to the specialty hospital (eTable 5 in the Supplement).

### Facilitators to Travel to a Specialty Hospital

**Figure 3.**
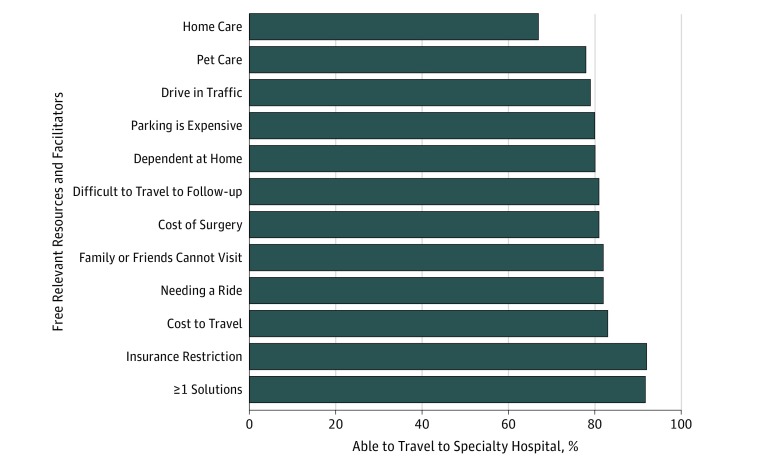
Impact of Facilitators on Respondents With Related Barriers The percentage of restricted respondents who could travel to the specialty hospital if allocated the relevant resource or facilitator for free.

Respondents were asked to determine which specific resources, if provided for free, would enable them to travel to the specialty hospital (facilitators). To clarify the effect of each facilitator, the ability to travel was examined within the subset of respondents who indicated they were restricted in a way that could logically be addressed by each facilitator (Figure 3). For example, the subset of respondents who indicated they were restricted by transportation-related barriers were reanalyzed in the presence of a free ride as a facilitator. Of the 752 individuals who identified at least 1 barrier preventing them from realigning, 94% (95% CI, 92%-96%) indicated that they would be enabled to travel to a specialty hospital if provided 1 or more of the facilitators. Of the 2879 total barriers acknowledged by 752 respondents, 1197 of the barriers (42%) were resolved with solutions of relatively low cost (eg, free rides, parking, pet care, and lodging for visitors).

## Discussion

This nationally representative survey suggests that over 90% of the US public could be motivated to travel an additional hour for complex cancer surgery if informed of a safety or quality advantage over their local hospital. This finding is in line with 2 previous surveys, which demonstrated that approximately 50% of patients in the United States were willing to travel longer than 5 hours for a reduction in mortality^16^ and that, of 214 patients presenting for cancer surgery at a university hospital, nearly half had traveled more than 2 hours to see an experienced surgeon.^17^ However, our findings contradict a previous study of patients at a Veterans Affairs hospital in Vermont, in which patients preferred to stay at their local hospital even if the operative mortality was double that of a regional hospital.^18^ The present study extends this previous work by reaching a large, nationally representative population instead of small distinct populations (veterans in Vermont or patients with cancer presenting to a university hospital). Additionally, the present study assesses willingness to travel an additional hour, which is relevant to the current distribution of centralized regional hospitals. An analysis of Medicare beneficiaries showed that only approximately 10% of patients would have to travel more than an additional hour from their local hospital to a centralized regional hospital for pancreatic resection or esophagectomy to comply with minimum volume standards.^7^

The majority of respondents indicated they would be motivated by quality and safety differentials currently known to exist. More specifically, a previous study in the National Cancer Database suggests the differential in lethal complication rates between the least safe and safest hospitals in the United States is 11.5% (95% CI, 9.3%-13.8%) for esophagectomy, and 14.3% (95% CI, 11.7%-16.9%) for gastrectomy.^6^ Based on data from this analysis, informing patients of this magnitude of advantage in surgical mortality would motivate 80% to 90% of patients to travel to a specialty hospital for these procedures.

The magnitude of care advantage that would motivate respondents to realign was variable. One-third of respondents in the current study were motivated by very subtle safety or quality advantages (<1%), while the remaining communicated a wide range of motivating differentials. Interestingly, most respondents identified different thresholds for each of the 6 quality and safety metrics that would motivate them to travel. In fact, 45% of respondents who were considered easily motivated for 1 metric (eg, would travel if there was <1% reduction in mortality) were in the most resistant subgroup in terms of another care attribute (eg, only would travel if the cure rate was >20% higher at specialty hospital). This metric-specific aspect to motivation suggests that efforts to encourage patients to travel to appropriate hospitals through patient education could potentially be enhanced by including multiple safety or quality end points.

A considerable proportion of respondents indicated a preference for the specialty hospital even in scenarios in which the specialty hospital offered minimal to no advantage in quality and safety. A free-text response option was included to understand the motivators within this subset of respondents (eAppendix 2 in the Supplement). Although the actual responses were quite diverse, thematically the responses implied an element of care being superior at the specialty hospital outside of the listed safety and quality motivators.

Despite a willingness to attend a regional specialty hospital for cancer surgery, most respondents (74%) acknowledged a specific barrier restricting them from doing so. This could be a contributing factor to the underwhelming extent to which spontaneous regionalization of complex cancer surgery has taken place in the United States.^19^ The barriers assessed in this survey include themes related to life disruption, transportation, lack of socioeconomic resources, and social support, which are consistent with other reported surveys.^16^ The belief that insurance status would restrict care access at a specialty hospital for surgery was common (53%), which is consistent with a previous qualitative analysis.^16^ Concern over insurance coverage appears to be justified for patients with cancer, as the current trend of narrowing insurance networks to lower costs disproportionately excludes cancer specialists at the National Cancer Institute or the National Comprehensive Cancer Network Centers.^20^ Furthermore, reports of patients being burdened with prohibitively high costs for out-of-network surgery have become common in the popular media, potentially affecting patient motivation to seek care at a new hospital.^16,21,22^

Although much of the surveyed population was restricted from traveling by specific barriers, most restricted respondents would be enabled to travel to a specialty hospital if provided with a facilitating resource (67%-92%, depending on the barrier). A large proportion of the facilitators could likely be provided at relatively low cost to the health care system (42%; free transportation, parking, pet care, lodging for visitors). We believe these low-cost interventions could enable a significant proportion of patients currently restricted from traveling to seek care at a hospital that is best prepared to care for them safely.

A set of preliminary analyses was performed to identify the subset of people who might be disproportionately refractory to traveling to a regional specialty hospital. Lower income, minority ethnicity, and living in a metropolitan area were found to be associated with resistance to leave their local hospital for improvements in outcomes. This may contribute to observed socioeconomic differences in attempts at regionalization of care for complex surgery, as previous studies have shown an association between nonwhite race, low income, and the tendency to receive cancer surgery at a low-volume hospital.^23,24^ Female participants were more likely to acknowledge a greater number of specific barriers in this survey. This may be in part due to the greater burden of dependent, elder, and home care that women disproportionately incur.^25^ Further efforts to encourage patient travel to appropriate hospitals may need to focus additional resources on specific subgroups.

### Limitations

While this survey aimed to identify challenges for individuals to travel to regional hospitals, there are many reasons besides travel itself for patients to stay at their local hospital. Possible other reasons include positive impressions of their local hospital, family concerns, follow-up concerns, expenses, and insurance, and these only were partially explored in this survey. Additionally, an important limitation to the current study is the fact that respondents were not necessarily patients, but a representative sample of the population at large. As a result, the impressions and sentiments that were communicated in the survey may differ from individuals who are actually facing these specific care decisions. However, there is some evidence that popular impressions mirror those of patients. A national survey done in the United Kingdom found that the preferences of patients with cancer are similar to the preferences of the public as a whole.^23^ Along the same lines, in the current study responses of individuals who were experienced health care consumers (ie, had a history of cancer or had received surgery) were similar to those who were not, although there were also some interesting minor differences (eTable 6 in the Supplement). For example, although patients with a history of cancer only were slightly less likely to be motivated to travel to the specialty hospitals than the general public (89% vs 95%), they tended to require a greater advantage in outcome to travel (approximately twice as likely to be resistant). It is possible that these individuals were influenced by prior experiences (positive or negative) with smaller or larger hospitals.

## Conclusions

It appears that much of the US public could be motivated to travel to safer hospitals for complex cancer surgery, yet the majority would require some support to do so. Further efforts to ensure that benefits from regionalization are equitable across sociodemographic strata are indicated.

## Notes

Supplement. **eAppendix 1.** Survey Questionnaire **eTable 1.** Unweighted and Weighted Demographics of Respondents **eTable 2.** Characteristics of Responders vs Nonresponders **eTable 3.** Distribution of Respondents Who Would Stay at Their Local Hospital vs Travel to a Specialty Hospital One Hour Away for Specific Improvements in Outcomes **eTable 4.** Multivariable Logistic Regression Analysis of Demographics Associated With Respondent’s Resistant to Moving to a Specialty Hospital for An Improvement in Outcomes **eTable 5.** Multivariable Logistic Regression Analysis of Demographics Associated With Having 3 or More Barriers (restricted cohort) **eAppendix 2.** Short Answer Responses From the Respondents Who Would Travel to the Specialty Hospital Despite Minimal Quantitative Benefit **eTable 6.** Subset Analysis of Respondents With a History of Cancer or Prior Surgery **eFigure 1.** Thresholds to Travel (increase in cure rate) **eFigure 2.** Thresholds to Travel (reduction in infection rate)
