# Supplementary material for: Motivators, Barriers, and Facilitators to Traveling to the Safest Hospitals in the United States for Complex Cancer Surgery
Source: JAMA Netw Open. 2018 Nov 16;1(7):e184595. doi: 10.1001/jamanetworkopen.2018.4595 (PMC6324377; doi:10.1001/jamanetworkopen.2018.4595)
Supplement: Supplement. — eAppendix 1. Survey Questionnaire eTable 1. Unweighted and Weighted Demographics of Respondents eTable 2. Characteristics of Responders vs Nonresponders eTable 3. Distribution of Respondents Who Would Stay at Their Local Hospital vs Travel to a Specialty Hospital One Hour Away for Specific Improvements in Outcomes eTable 4. Multivariable Logistic Regression Analysis of Demographics Associated With Respondent’s Resistant to Moving to a Specialty Hospital for An Improvement in Outcomes eTable 5. Multivariable Logistic Regression Analysis of Demographics Associated With Having 3 or More Barriers (restricted cohort) eAppendix 2. Short Answer Responses From the Respondents Who Would Travel to the Specialty Hospital Despite Minimal Quantitative Benefit eTable 6. Subset Analysis of Respondents With a History of Cancer or Prior Surgery eFigure 1. Thresholds to Travel (increase in cure rate) eFigure 2. Thresholds to Travel (reduction in infection rate) [file jamanetwopen-1-e184595-s001.pdf]

Supplementary Online Content

Resio BJ, Chiu AS, Hoag JR, et al. Motivators, barriers, and facilitators to traveling to the safest hospitals in the United States for complex cancer surgery. *JAMA Netw Open*. 2018;1(7):e184595. doi:10.1001/jamanetworkopen.2018.4595

eAppendix 1. Survey Questionnaire
eTable 1. Unweighted and Weighted Demographics of Respondents
eTable 2. Characteristics of Responders vs Nonresponders
eTable 3. Distribution of Respondents Who Would Stay at Their Local Hospital vs Travel to a Specialty Hospital One Hour Away for Specific Improvements in Outcomes
eTable 4. Multivariable Logistic Regression Analysis of Demographics Associated With Respondent's Resistant to Moving to a Specialty Hospital for An Improvement in Outcomes
eTable 5. Multivariable Logistic Regression Analysis of Demographics Associated With Having 3 or More Barriers (restricted cohort)
eAppendix 2. Short Answer Responses From the Respondents Who Would Travel to the Specialty Hospital Despite Minimal Quantitative Benefit
eTable 6. Subset Analysis of Respondents With a History of Cancer or Prior Surgery
eFigure 1. Thresholds to Travel (increase in cure rate)
eFigure 2. Thresholds to Travel (reduction in infection rate)

This supplementary material has been provided by the authors to give readers additional information about their work.

© 2018 Resio BJ et al. *JAMA Network Open*.

eAppendix 1. Survey Questionnaire

Base: all respondents

Demographic Question (Select all that apply)
Which of the following things are true, if any?

1. I make my own medical decisions
2. I have been diagnosed with cancer in the past
3. I have had surgery in the past
4. None of these are true of me

Base: all respondents

Q01

How long does it currently take you to get to the hospital you use most often?
(If you have not gone to a hospital recently, how long would it take for you to get to a hospital from where you live?)

___ (hours) ___ (minutes)

Base: all respondents

Imagine you have been diagnosed with cancer. A complex surgery is the best way to cure your cancer. Your choices for surgery are a smaller hospital close to home, and a hospital that specializes in cancer surgery that is 1 hour farther away than your local hospital. It is unclear if safety is the same at both hospitals (the chances of dying after surgery may be higher at one hospital than the other).

Base: Total respondents

Q03a

Which hospital would you choose?

Your Local Hospital	Specialty Hospital (1 hour farther away)
Chances of dying from surgery 10%	Chances of dying from surgery 10%

© 2018 Resio BJ et al. *JAMA Network Open*.

Base: Q3a=1 (local)

Q03b

Which hospital would you choose?

Your Local Hospital	Specialty Hospital (1 hour farther away)
Chances of dying from surgery<br>10%	Chances of dying from surgery<br>9%

Base: Q3b=1 (local)

Q03c

Which hospital would you choose?

Your Local Hospital	Specialty Hospital (1 hour farther away)
Chances of dying from surgery<br>10%	Chances of dying from surgery<br>7%

Base: Q3c=1 (local)

Q03d

Which hospital would you choose?

Your Local Hospital	Specialty Hospital (1 hour farther away)
Chances of dying from surgery<br>10%	Chances of dying from surgery<br>5%

Base: Q3d=1 (local)

Q03e

Which hospital would you choose?

Your Local Hospital	Specialty Hospital (1 hour farther away)
Chances of dying from surgery<br>10%	Chances of dying from surgery<br>3%

© 2018 Resio BJ et al. *JAMA Network Open*.

Base: Q3e=1 (local)

Q03f

Which hospital would you choose?

Your Local Hospital	Specialty Hospital (1 hour farther away)
Chances of dying from surgery<br>10%	Chances of dying from surgery<br>**Less than 1%**

Base: all respondents

Q04

Even if you thought surgery was better at the specialty hospital, which of the following things (if any) would prevent you from travelling 1 hour to the specialty hospital for surgery?

Statements in row [RANDOMIZE AND RECORD ORDER OF THE STATEMENTS]:

1. Needing a ride to travel an hour
2. Having to drive myself in traffic
3. Parking is expensive at the specialty hospital
4. Having enough money to pay to travel an hour (hire a taxi, pay for bus or subway)
5. I need to be home to take care of somebody
6. I need to be home to take care of a pet
7. I need to be home to take care of a business I own
8. I need to be home to take care of my house or apartment (pay bills, yard work)
9. My family and friends would have difficulty visiting me at a farther hospital
10. Surgery at the specialty hospital would cost more money
11. My insurance restricts me from going to a specialty hospital
12. My work won’t let me get that much time off
13. I would have a hard time going back and forth to see doctors after surgery

Answers in column:

1. Would prevent me from traveling an hour
2. Would not prevent me

Base: all respondents

Q05[Grid,S per row]

If you thought surgery was better at the specialty hospital, if given to you for free, which of the following things (if any) would make it possible for you to travel 1 hour to the specialty hospital for surgery?

© 2018 Resio BJ et al. *JAMA Network Open*.

Statements in row [RANDOMIZE AND RECORD ORDER OF THE STATEMENTS]:

1. Free rides to any hospital
2. Free help caring for somebody that lives with you
3. Free help caring for a pet that lives with you
4. Free help taking care of your house or apartment
5. Free rides for your friends and family to visit you at any hospital
6. A free place for your family to stay close to the specialty hospital
7. Surgery at both hospitals costs the same amount of money
8. My insurance will pay for surgery at any hospital I want to go to

Answers in column:

1. I would travel to the specialty hospital
2. I would stay at the local hospital

Base: all respondents

Q06

Several organizations are trying to make information about hospitals and surgeons easier for patients to see. Nobody knows if this information is helpful to patients. What information (if any) would cause you to travel 1 hour to have surgery at a specialty hospital (instead of having surgery at a local hospital close to home)?

Statements in row [RANDOMIZE AND RECORD ORDER OF THE STATEMENTS]:

1. The surgeon at the specialty hospital is safer (fewer patients die after surgery)
2. The surgeon at the specialty hospital is more likely to get all of the cancer out (not leave cancer behind)
3. Patients at the speciality hospital have lower chances of getting an infection after surgery
4. Patients at the specialty hospital are less likely to have complications after surgery
5. The specialty hospital performs more of your needed surgeries
6. Patients at the specialty hospital are more likely to be cured

Answers in column:

1. I would likely go to the specialty hospital
2. I would stay at the local hospital

© 2018 Resio BJ et al. *JAMA Network Open*.

Base: [SHOW IF Q06_1=1]

Q06a1 [Q, RANGE 0-15]

You told us you would go to the specialty hospital if the surgeon at the specialty hospital is safer (fewer patients die after surgery).

Tell us how much safer the specialty surgeon would need to be, for you to go the specialty hospital.

"If 15% of people die after surgery with the local surgeon, I would only go the specialty surgeon if his or her rate of patients dying was less than [NUMBOX, RANGE 0-15]%."

Base: [SHOW IF Q06_2=1]

Q06a2 [Q, RANGE 80-100]

You told us you would go to the specialty hospital if surgery at the specialty hospital is more likely to get all of the cancer out (not leave cancer behind).

Tell us how much better the specialty hospital would need to be, for you to go the specialty hospital.

"If 80% of patients of people have all their cancer removed at the local hospital, I would only go the specialty hospital if more than [NUMBOX, RANGE 80-100]% of patients had all their cancer removed."

Base: [SHOW IF Q06_3=1]

Q06a3 [Q, RANGE 0-20]

You told us you would go to the specialty hospital if the specialty hospital has lower chances of getting an infection after surgery.

Tell us how much safer the specialty hospital would need to be, for you to go the specialty hospital.

"If 20% of the patients at the local hospital get an infection in the hospital, I would only go to the specialty hospital if their rates were lower than [NUMBOX, RANGE 0-20]%."

Base: [SHOW IF Q06_4=1]

Q06a4 [Q, RANGE 0-40]

You told us you would go to the specialty hospital if the specialty hospital has fewer complications after surgery.

Tell us how much safer the specialty hospital would need to be, for you to go the specialty hospital.

"If 40% of the patients at the local hospital have a complication after surgery, I would only go to the specialty hospital if the rate of complications was less than [NUMBOX, RANGE 0-40]%."

© 2018 Resio BJ et al. *JAMA Network Open*.

eTable 1. Unweighted and Weighted Demographics of Respondents

	Number of Respondents	Percent of Respondents	Weighted Percent of Respondents
Sex			
Male	525	51.7	48.4
Female	491	48.3	51.6
Age			
18-29	167	16.4	21.1
30-44	210	20.7	25.0
45-59	277	27.3	26.0
60+	362	35.6	27.9
Race/ Ethnicity			
White, Non-Hispanic	716	70.5	64.0
Black, Non-Hispanic	108	10.6	11.8
Other, Non-Hispanic	47	4.6	4.8
Hispanic	112	11.0	15.9
2+ Races, Non-Hispanic	33	3.3	3.5
Annual Household Income			
<$30000	174	17.1	19.2
$30-60k	228	22.4	22.9
$60-100k	321	31.6	23.4
$100-200k	299	29.4	28.2
>200K	69	6.8	3.4
Urban/ Rural			
Non-Metro	155	15.3	14.6
Metro	861	84.7	85.4
US Region			
Northeast	165	16.2	17.8
Midwest	241	23.7	20.8
South	367	36.1	37.7
West	243	23.9	23.7
Education level			
Less than high school	74	7.3	9.6
High school	255	25.1	30.3
Some college	292	28.7	28.6
Bachelor's degree or higher	395	38.9	31.5
Had Major Surgery in the Past			
Yes	512	49.6	46.5
No	504	50.4	53.6

© 2018 Resio BJ et al. *JAMA Network Open.*

Had Cancer in the Past			
Yes	89	8.8	7.5
No	927	91.2	92.6
Makes Own Medical Decisions			
Yes	848	83.5	82.2
No	168	16.5	17.8

© 2018 Resio BJ et al. *JAMA Network Open*.

eTable 2. Characteristics of Responders vs Nonresponders

	Responders (n=1016)		Non- responders (n=792)		P Value
	#	%	#	%	
Sex					
Male	525	51.7%	361	45.6%	0.01
Female	491	48.3%	431	54.4%	0.01
Age					
18-29	167	16.4%	214	27.0%	<.001
30-44	210	20.7%	232	29.3%	<.001
45-59	277	27.3%	209	26.4%	0.67
60+	362	35.6%	127	16.0%	<.001
Race/ Ethnicity					
White, Non-Hispanic	716	70.5%	463	58.5%	<.001
Black, Non-Hispanic	108	10.6%	128	16.2%	<.001
Other, Non-Hispanic	47	4.6%	35	4.4%	0.83
Hispanic	112	11.0%	136	17.2%	<.001
2+ Races, Non-Hispanic	33	3.2%	30	3.8%	0.53
Annual Household Income					
<$30,000	174	17.1%	175	22.1%	0.008
$30,000-59,999	228	22.4%	206	26.0%	0.08
$60,000-99,999	321	31.6%	177	22.3%	<.001
$100,000-200,000	299	29.4%	182	23.0%	0.002
>$200,000	69	6.8%	52	6.6%	0.85
Urban/ Rural					
Non-Metro	155	15.3%	110	13.9%	0.41
Metro	861	84.7%	682	86.1%	0.41
US Region					
Northeast	165	16.2%	158	19.9%	0.04
Midwest	241	23.7%	137	17.3%	<.001
South	367	36.1%	322	40.7%	0.05
West	243	23.9%	175	22.1%	0.36
Education level					
Less than high school	74	7.3%	91	11.5%	0.002
High school	255	25.1%	256	32.3%	<.001
Some college	292	28.7%	230	29.0%	0.89

© 2018 Resio BJ et al. *JAMA Network Open*.

Bachelor's degree or higher	395	38.9%	215	27.1%	<.001

© 2018 Resio BJ et al. *JAMA Network Open*.

eTable 3. Distribution of Respondents Who Would Stay at Their Local Hospital vs Travel to a Specialty Hospital One Hour Away for Specific Improvements in Outcomes

Prompt	Stay at local Hospital, N (%)*	Travel to Specialty Hospital, N (%)*
Patients at the specialty hospital have lower chances of getting an infection after surgery	87 (8.6)	929 (91.4)
Patients at the specialty hospital are more likely to be cured	81 (8.0)	935 (92.0)
Patients at the specialty hospital are less likely to have complications after surgery	78 (7.7)	938 (92.3)
The surgeon at the specialty hospital is safer (fewer patients die after surgery)	82 (8.1)	934 (91.9)
The surgeon at the specialty hospital is more likely to get all of the cancer out (not leave cancer behind)	80 (7.9)	935 (92.1)
The specialty hospital performs more of your needed surgeries	93 (9.2)	922 (90.7)

*Frequencies and percentages are weighted to be nationally representative.

© 2018 Resio BJ et al. *JAMA Network Open*.

eTable 4. Multivariable Logistic Regression Analysis of Demographics Associated With Respondent's Resistant to Moving to a Specialty Hospital for An Improvement in Outcomes

Effect	N	Adjusted* Odds Ratio	95% Confidence Limits		P Value
High school vs college education	329 vs. 687	1.09	0.71	1.70	0.69
Metro vs non-metro	861 vs. 155	2.08	1.01	4.28	0.05
Age <36 vs 36-50	254 vs. 201	0.53	0.28	0.99	0.04
Age 51-65 vs 35-50	331 vs. 201	1.20	0.70	2.07	0.10
Age 66-80 vs 35-50	200 vs. 201	0.99	0.52	1.87	0.57
Age >80 vs 35-50	30 vs. 201	0.77	0.21	2.86	0.82
Race non-white vs. white	300 vs. 716	1.60	1.05	2.42	0.03
Income <25k vs >25K	133 vs. 883	2.01	1.19	3.39	0.01
Female vs Male	491 vs. 525	1.49	0.99	2.23	0.06
Does not make medical decisions vs does	168 vs. 848	1.38	0.83	2.27	0.21
Cancer in the past vs no cancer	89 vs. 927	2.07	1.05	4.07	0.03
No surgery in past vs yes	504 vs. 512	1.94	1.23	3.06	0.00
Time to local hospital <1hr vs >1hr	989 vs. 27	0.66	0.23	1.88	0.44

*Model is adjusted for education, residence in a metropolitan statistical area, age, ethnicity, income, sex, previous surgery or cancer diagnosis, and travel time from one's local hospital

© 2018 Resio BJ et al. *JAMA Network Open*.

eTable 5. Multivariable Logistic Regression Analysis of Demographics Associated With Having 3 or More Barriers (restricted cohort)

Effect	N	Adjusted* Odds Ratio	95% Confidence Limits		P Value
High school Vs College Education	329 vs. 687	1.33	0.98	1.80	0.07
Metro vs Non-Metro	861 vs. 155	1.34	0.89	2.02	0.16
age <36 vs 36-50	254 vs. 201	1.17	0.79	1.75	0.01
age 51-65 vs 35-50	331 vs. 201	0.68	0.46	1.01	0.31
age 66-80 vs 35-50	200 vs. 201	0.69	0.44	1.09	0.46
age >80 vs 35-50	30 vs. 201	0.54	0.22	1.35	0.29
Race Non-white vs. White	300 vs. 716	0.76	0.56	1.03	0.07
Income <25k vs >25K	133 vs. 883	2.90	1.94	4.33	<.001
Female vs Male	491 vs. 525	1.66	1.25	2.20	<.001
Does not make medical decisions vs Does	168 vs. 848	1.07	0.73	1.57	0.72
No Cancer in the past vs Yes	89 vs. 927	1.21	0.70	2.11	0.49
No Surgery in past vs Yes	504 vs. 512	1.08	0.80	1.47	0.61
Time to local hospital <1hr vs >1hr	989 vs. 27	1.00	0.42	2.37	0.99

*Model is adjusted for education, residence in a metropolitan statistical area, age, ethnicity, income, sex, previous surgery or cancer diagnosis, and travel time from one's local hospital

eAppendix 2. Short Answer Responses From the Respondents Who Would Travel to the Specialty Hospital Despite Minimal Quantitative Benefit

**Is there any other information about the differences between specialty hospitals and local hospitals that would make you go to the specialty hospital? (only answered by 47/160 of these respondents)**
Bedside manner and not as many forms to fill
Better care during and after surgery.
Better reputation of the doctors, better transparency in costs.
Cleaner, better organized, faster response time, reputation, choice of Doctors
Given the previous safety concerns are equal, I would be MUCH more inclined if at all possible to go to a specialty hospital just because there are so many other factors specific to a type of condition that they should be better prepared to deal with.
How well they treat patients and their families. Our local hospital is very good with patient relations and amenities. However, for services requiring specialization, my wife and I have learned that university based hospitals like University of ***
I am a patient of *** Hospital and I would recommend it to anybody.
I believe the specialty hospital would have the most up-to-date research available to draw on, and if the case calls for it perhaps be more inclined to try treatments that are new.
I will always go with a place that specializes in a certain treatment, it is common sense to me.
I would ALWAYS go to the specialty hospital
I would always choose the specialty hospital because I believe that they would be more informed and better equipped to handle my problem.
I would always choose the specialty hospital no matter the cost or travel since there is a better outcome there.
I would choose the specialty hospital, because that's there area of expertise.
I would go to a specialty hospital no matter what the circumstances. I feel that the staff at a specialty hospital should have the experience and equipment to do a better job and should have seen more cases of cancer than a local hospital.
I would go to the specialty hospital for the experience of the surgeon alone, even if all other factors were the same as the local hospital.
I would go to the specialty hospital no matter what.
I would go where my doc tells me to go.
I would not need to travel one hour further to a specialty hospital, as these types of facilities are fairly close to my home.
I'm pretty sure I said I would go the specialty hospital, but you kept asking me questions like I said I'd use the local hospital. 1 hour drive is worth any percentage better care/less risk to me.
If there was a complication with the surgery or the recovery, the specialty hospital would likely be more equipped to handle it.

© 2018 Resio BJ et al. *JAMA Network Open*.

Just if the doctors are more qualified and can grantee that it is better or I have more chance of beating what i have there than at the other hospital
Just the fact that by being a specialty hospital, I feel that the staff would be more qualified to take care of me if I had that condition.
**Is there any other information about the differences between specialty hospitals and local hospitals that would make you go to the specialty hospital? (only answered by 47/160 of these respondents)**
More advanced treatments or techniques. More experience and better equipment for the particular cancer.
More logical plans of treatment.
More qualified staff will be at a specialty hospital and the staff is prepared for an emergency if something should happened during surgery
My local hospitals are aweful. Most of the time we travel 2hrs for anything serious.
No matter what, I will still choose a specialty to treat me.
Overall comfort for me and my family.
Quality of the surgeon and staff.
Reviews from others who have had the same surgery performed at the special hospital.
Specialities just seem more in tune with specific needs because they deal with so many patients in that area. In the same way I would prefer to go to a birthing center rather than a general hospital, I would prefer to go to a specialty cancer facility tha
Specialty hospitals typically have more experienced doctors and are focused on only one specialty, so I would trust their expertise more than a general practitioner/surgeon.
Statistics...just give me statistics.
Success rates are more common as the number of surgeries performed rises. A surgeon at a specialty hospital is likely to have performed more such relevant surgeries than a surgeon at a local hospital.
The doctors are more up to date with current treatments. They also have subspecialty/specialty conferences (tumor boards) where individual cases are discussed to mutliple oncology specialities. This way they can add their input. The speciality hospitals a
These answers are kind of meaningless in my case, since I would definitely go to a specialty hospital if I had a complicated surgery to do. I don't need convincing or help - I would just go to a hospital that specializes in my type of surgery. To me, a
What is up with the free rides, free pet care, free whatever else nonsense questions? They don't apply to 98% of people!!!!!!
Would they also do rehab or reconstructive surgery?
depends on protocals
type of cancer and treatment options
good service, good hospitality and good utility
if the specialty hospital was rated highly, such as being in the top ten of the country.
its an unfair question - there's comfort level with surgeon to consider, nurses, after care - its just not a percentage type scenario

© 2018 Resio BJ et al. *JAMA Network Open*.

locally where I live the specialty hospital has no cases of mersa. All other hospitals in the area have had several cases of mersa. Patients are dying because of the mersa not the problem they went in for.
reputation of the specialty hospital vs. the local
word of mouth, previous patient experiences....news/investigational news reports.......3rd party rankings, etc.
yes, the specialty hospital specializes in that field of care

© 2018 Resio BJ et al. *JAMA Network Open*.

eTable 6. Subset Analysis of Respondents With a History of Cancer or Prior Surgery

**Motivators**	**Healthcare experienced (n=472)**	**Healthcare naïve (n=544)**	**P Value**
	**n (%) motivated**	**n (%) motivated**	
Patients at the specialty hospital have lower chances of getting an infection after surgery	445 (94)	484 (89)	0.002
Patients at the specialty hospital are more likely to be cured	450 (95)	485 (89)	<0.001
Patients at the specialty hospital are less likely to have complications after surgery	450 (95)	488 (90)	<0.001
The surgeon at the specialty hospital is safer (fewer patients die after surgery)	450 (95)	484 (89)	<0.001
The surgeon at the specialty hospital is more likely to get all of the cancer out (not leave cancer behind)	450 (95)	486 (89)	<0.001
The specialty hospital performs more of your needed surgeries	446 (95)	476 (88)	<0.001
Overall motivators	449 (95)	484 (89)	<0.001
**Barriers**			
Percent with barriers	358 (76)	398 (73)	0.3279
**Facilitators**			
Percent with barriers who would travel with one or more corresponding solutions	447 (93)	508 (91)	0.3767

© 2018 Resio BJ et al. *JAMA Network Open*.

eFigure 1. Thresholds to Travel (increase in cure rate)

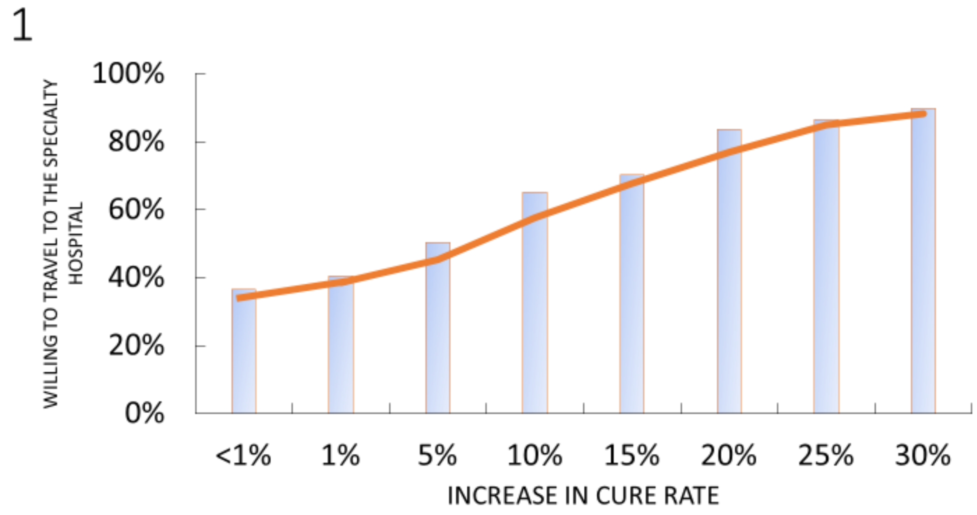

The percentage of respondents who would travel to the specialty hospital is shown (y-axis), across a range of margins by which the specialty hospital was superior to the local hospital (x-axis). A separate by graph is shown for the remaining 2 of 6 quality and safety indicators including 1) increase in cure rate, 2) Reduction in infection rate. Each bar represents the total percentage that would travel at any given threshold (and therefore includes respondents who indicated a specific threshold or any smaller margin).

© 2018 Resio BJ et al. *JAMA Network Open*.

eFigure 2. Thresholds to Travel (reduction in infection rate)

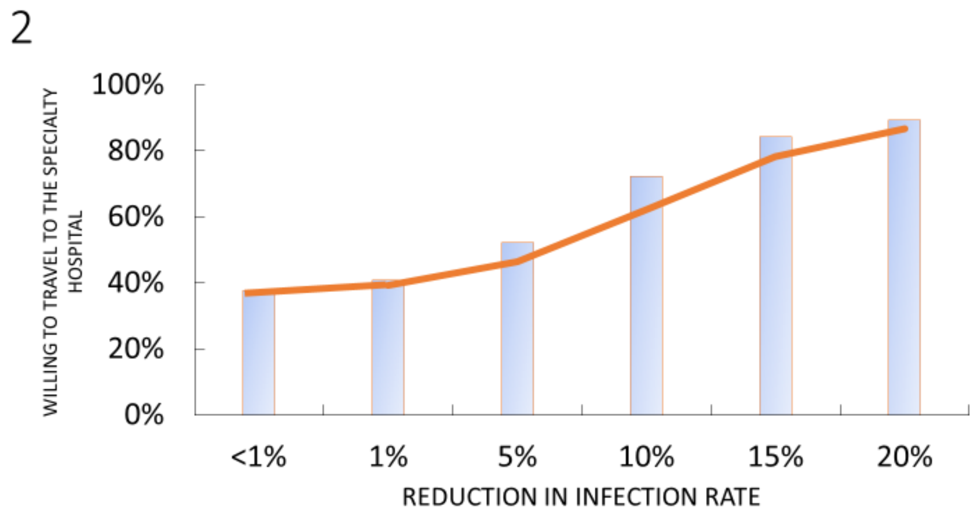

The percentage of respondents who would travel to the specialty hospital is shown (y-axis), across a range of margins by which the specialty hospital was superior to the local hospital (x-axis). A separate by graph is shown for the remaining 2 of 6 quality and safety indicators including 1) increase in cure rate, 2) Reduction in infection rate. Each bar represents the total percentage that would travel at any given threshold (and therefore includes respondents who indicated a specific threshold or any smaller margin).

© 2018 Resio BJ et al. *JAMA Network Open*.
